# Serum BDNF Levels Are Reduced in Patients with Disorders of Consciousness and Are Not Modified by Verticalization with Robot-Assisted Lower-Limb Training

**DOI:** 10.1155/2020/5608145

**Published:** 2020-05-22

**Authors:** Sergio Bagnato, Giuseppe Galardi, Francesco Ribaudo, Cristina Boccagni, Teresa Valentina Fiorilla, Francesca Rubino, Maria Enza D'Ippolito, Maria Andriolo

**Affiliations:** ^1^Unit of Neurophysiology and Unit for Severe Acquired Brain Injuries, Rehabilitation Department, Giuseppe Giglio Foundation, Cefalù, Italy; ^2^Neurorehabilitation Department, IRCCS Neuromed, Pozzilli, Italy; ^3^Clinical Pathology and Microbiology Laboratory, Giuseppe Giglio Foundation, Cefalù, Italy

## Abstract

Little is known about plastic changes occurring in the brains of patients with severe disorders of consciousness (DOCs) caused by acute brain injuries at rest and during rehabilitative treatment. Brain-derived neurotrophic factor (BDNF) is a neurotrophin involved in neurogenesis and synaptic plasticity whose production is powerfully modulated by physical exercise. In this study, we compared serum BDNF levels in 18 patients with unresponsive wakefulness syndrome (UWS) and in a minimally conscious state (MCS) with those in 16 sex- and age-matched healthy controls. In 12 patients, serum BDNF levels before and after verticalization with ErigoPro robot-assisted lower-limb training were compared. Serum BDNF levels were significantly lower in patients (median, 1141 pg/ml; 25^th^ and 75^th^ percentiles, 1016 and 1704 pg/ml) than in controls (median, 2450 pg/ml; 25^th^ and 75^th^ percentiles, 2100 and 2875 pg/ml; *p* < 0.001). BDNF levels measured before and after verticalization with robot-assisted lower-limb training did not change (*p* = 0.5). Moreover, BDNF levels did not differ between patients with UWS and MCS (*p* = 0.2), or between patients with traumatic and nontraumatic brain injuries (*p* = 0.6). BDNF level correlated positively with the time since brain injury (*p* = 0.025). In conclusion, serum BDNF levels are reduced in patients with UWS and MCS and cannot be improved by verticalization associated with passive lower-limb training. Additional studies are needed to better understand the mechanisms underlying BDNF reduction in patients with DOCs and to determine the best rehabilitative strategies to promote restorative plastic changes in these patients.

## 1. Introduction

To foster the recovery of consciousness in patients in a vegetative state or in a minimally conscious state (MCS) after acute brain injury is among the most challenging tasks in modern rehabilitation. The vegetative state, also referred to as unresponsive wakefulness syndrome (UWS) [1, 2], is a disorder of consciousness (DOC) in which the patient shows no sign of awareness of him- or herself and the external world. The MCS, which may arise directly from acute brain injury or represent the evolution of UWS, is a condition in which the patient shows minimal and fluctuating signs of awareness [3]. UWS and MCS are the results of acute and massive disruption of the brain's ability to subtend normal consciousness; depending on the severity of the brain damage, they may last indefinitely or improve gradually until the patient recovers normal consciousness. Although the mechanisms leading to the recovery of consciousness in patients with DOCs are largely unknown, they necessarily involve plastic changes in the brain, i.e., structural and functional modifications in the circuits subtending the consciousness [4].

Brain-derived neurotrophic factor (BDNF) is a neurotrophin that plays a key role in brain plasticity, promoting structural and functional changes in the brain such as neuro- and synaptogenesis, short- and long-lasting changes in synaptic activity, and the development of neural circuits involved in memory and cognitive functions [5–7]. BDNF expression changes after acute brain injury, suggesting its potential role in neuronal and synaptic reorganization following brain damage. In animal models of traumatic brain injury (TBI), BDNF expression is upregulated in the hippocampus and cerebral cortex [8, 9]; peripheral levels of BDNF after acute brain injury in humans have been scarcely evaluated. Some studies suggest that the secretion of BDNF in the brain is reduced immediately after TBI and that this reduction is more evident in patients with poor outcomes [10, 11], whereas other studies have revealed increased BDNF levels [12]. Few studies have evaluated BDNF levels in patients with brain injuries other than TBI. For example, lower BDNF levels are associated with worse outcomes in patients with neonatal hypoxic-ischemic encephalopathy [13].

A very interesting property of BDNF, especially from a rehabilitative perspective, is that its expression can be upregulated by physical exercise. In healthy humans, short-term exercise increases the BDNF level in the bloodstream, at a magnitude that depends on the exercise intensity [14]. Moreover, the increase in circulating BDNF is more evident after prolonged exercise, possibly as a result of its release from the hippocampus, cortex, and cerebellum [15]. The positive effects of exercise on the BDNF level and cognitive functions have also been demonstrated in elderly patients with cognitive deficits, such as mild cognitive impairment [16]. Thus, the beneficial effects of exercise on cognitive functions may depend, at least in part, on BDNF-mediated mechanisms [17].

Patients with severe DOCs cannot benefit from the BDNF-mediated effects of active exercise because the majority of them cannot cooperate with rehabilitative treatment; only few patients in an MCS have a minimal ability to perform exercises voluntarily. Interestingly, some rehabilitative treatments that do not require active patient participation effectively improve the level of consciousness in patients with DOCs. For example, short periods of standing, achieved using a tilt table, improve arousal and awareness in patients with UWS and those in an MCS [18, 19], although they are associated with a high occurrence of orthostatic hypotension [19]. To reduce orthostatic intolerance, robotic systems that perform patient verticalization in association with passive step training have been developed. These systems, such as the ErigoPro robot (Hocoma, Volketswil, Switzerland), activate the venomuscular pumps of the lower limbs and significantly reduce the occurrence of hypotension in patients with DOCs [20]. However, the neurobiological mechanisms potentially activated by verticalization with robotic lower-limb training have not been investigated in patients with severe DOC.

This study had two main aims: to determine whether the baseline serum BDNF level of patients with DOCs differed from that of sex- and age-matched healthy subjects and to investigate whether the BDNF level can be modulated by verticalization with ErigoPro robot-assisted lower-limb training. The results of this study will aid our understanding of how BDNF expression is modulated after severe brain injury in patients with UWS and in an MCS and whether a rehabilitative treatment based on verticalization associated to passive lower limb robot-assisted training is effective in modifying BDNF levels.

## 2. Materials and Methods

### 2.1. Protocol Approval and Informed Consent

The study abided by the Declaration of Helsinki and was approved by the regional ethics review board (Palermo 1 Ethics Committee, Palermo, Italy). Patients' legal guardians and healthy subjects provided written informed consent to all study procedures.

### 2.2. Participants

Eighteen patients with UWS or in an MCS following acute brain injuries and 16 sex- and age-matched healthy controls participated in this study (see Table 1 for demographic and clinical details). Twelve patients also participated in the evaluation of BDNF levels after treatment with the ErigoPro robot; six patients were excluded from this treatment due to bone fractures (*n* = 3), the need for mechanical ventilation (*n* = 1), severe dystonic postures (*n* = 1), and psychomotor agitation (*n* = 1). All patients were admitted to our Unit for Severe Acquired Brain Injuries for intensive rehabilitation following acute brain injuries. Inclusion criteria for patients were as follows: (i) diagnosis of UWS or MCS according to the current diagnostic criteria [1, 3] at the time of study inclusion, (ii) study inclusion ≥ 1 month after acute brain injury, and (iii) age 18–65 years (patients aged >65 years were excluded due to the increased risk of neurodegenerative diseases). Exclusion criteria were as follows: (i) previous history of brain injury or psychiatric or neurodegenerative disease and (ii) coexisting neoplasia, severe organ dysfunction, or unstable clinical condition (e.g., hemodynamic instability or severe respiratory failure). Healthy controls were recruited among blood donors and had no previous history of neurological, psychiatric, or neoplastic disease.

The authors, experts in the clinical evaluation of patients with DOCs (SB, CB, and TVF), diagnosed UWS and MCSs based on assessment using the Coma Recovery Scale-Revised (CRS-R) [21]. The CRS-R, which provides criteria for the diagnosis of UWS, MCS, and emergence from MCS, is the most reliable tool available for the assessment of patients with DOCs following coma [22]. Diagnoses of UWS and MCS were accepted only when confirmed by clinical evaluations performed on 3 consecutive days, including those of blood sample collection.

### 2.3. Verticalization with Robot-Assisted Stepping

The ErigoPro system (Hocoma) consists of a tilt table with an integrated robotic stepping device that permits early verticalization of uncollaborative patients. The patient's upper body is secured by a harness that fixes the chest and pelvis to the table. The feet are secured to mobile footplates for computer-controlled leg movement. The physiotherapist determines the range of motion at the level of each hip before treatment, and this range is kept stable throughout the exercise period. The inclination of the tilt table can be adjusted gradually from horizontal to vertical. The speed of leg movement can be modified from 0 to 80 steps per minute.

In this study, the number of steps was fixed at 50 per minute and maintained throughout the duration of the experiment. Robot-assisted stepping commenced after the patient had been secured to the table in the horizontal position and lasted for 40 minutes. During the first 10 minutes of stepping, the table was inclined gradually from 0° to 45° at a rate of 15° every 2.5 minutes. Then, the table was inclined to 60° and maintained at this inclination for 30 minutes. These parameters were chosen because they are used most frequently in our center, and most patients tolerate them well. Patients' heart rates, blood pressure, and oxygen saturation were monitored throughout the ErigoPro treatment to prevent complications associated with orthostatism (e.g., hypotension and syncope).

### 2.4. BDNF Analysis

Blood samples were collected from all patients and healthy controls via venipuncture after ≥30-minute rest. For patients, blood samples were collected immediately before and after verticalization with robot-assisted training. Participants' blood samples were centrifuged at 1500 rpm for 15 minutes, and the serum was aliquoted and frozen at –80°C until analyzed using the Human BDNF PicoKine™ enzyme-linked immunosorbent assay kit (Booster Biological Technology, Pleasanton, CA, USA) according to the manufacturer's instructions. Briefly, this kit is a solid-phase immunoassay designed specifically for the measurement of human BDNF using a 96-well strip plate precoated with BDNF-specific antibody. Biotinylated, polyclonal, BDNF-specific goat antibody was used for detection, and monoclonal mouse antibody was used for capture. Concentrations were calculated to permit evaluation of the mean absorbance of each sample at 450 nm with a microplate reader. Results were determined via an instrument-specific calibration curve and are presented in pg/ml. The test sensitivity and detection range are <15 and 31.2–2000 pg/ml, respectively. Samples with BDNF values > 2000 pg/ml were further diluted and analyzed.

### 2.5. Statistical Analysis

Demographic and clinical data are expressed as means ± standard deviations; BDNF levels are expressed as medians and 25^th^ and 75^th^ percentiles. We used the Mann–Whitney *U* test to compare demographic characteristics and BDNF levels between patients with DOCs and controls. The same test was also used to compare patients' BDNF levels before and after verticalization and baseline BDNF levels between patients with different levels of consciousness (UWS and MCS) and DOC etiologies (traumatic and nontraumatic). Finally, we used Spearman's rank-correlation test to examine whether BDNF levels correlated with the time since brain injury. *p* values < 0.05 were considered to be significant.

## 3. Results

ErigoPro verticalization did not have adverse effects in any patient. Patients with severe DOCs did not differ from healthy controls in terms of age (*U* = 134, *p* = 0.7) or sex (Table 1).

**Figure 1:**
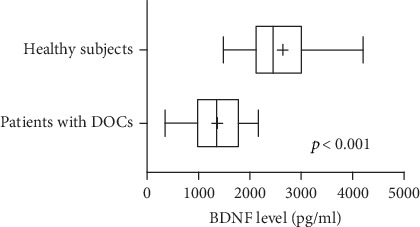
Serum BDNF levels in patients with DOCs and healthy controls. BDNF levels were significantly lower in patients with DOCs than in controls. Bars indicate ranges, boxes indicate 25^th^ and 75^th^ percentiles, lines in boxes indicate medians, and +s indicate means. BDNF: brain-derived neurotrophic factor; DOC: disorder of consciousness.

**Figure 2:**
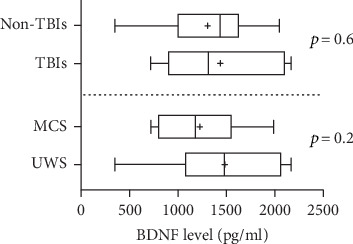
Serum BDNF levels in patients with different DOCs and brain injury etiologies. BDNF levels did not differ between patients with UWS and those in an MCS as well as between patients with TBIs and non-TBIs. Bars indicate ranges, boxes indicate 25^th^ and 75^th^ percentiles, lines in boxes indicate medians, and +s indicate means. BDNF: brain-derived neurotrophic factor; MCS: minimally conscious state; TBI: traumatic brain injury; UWS: unresponsive wakefulness syndrome.

**Figure 3:**
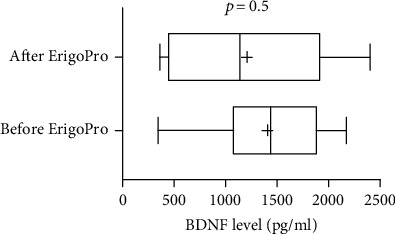
Serum BDNF levels before and after verticalization with robot-assisted lower-limb training. In patients with DOCs, BDNF levels did not differ before and after ErigoPro training. Bars indicate ranges, boxes indicate 25^th^ and 75^th^ percentiles, lines in boxes indicate medians, and +s indicate means. BDNF: brain-derived neurotrophic factor.

**Figure 4:**
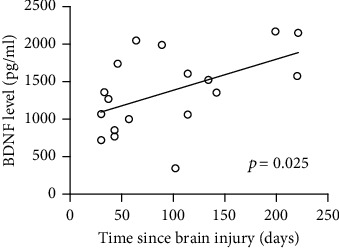
Correlations between BDNF levels and time since brain injury. Patients' BDNF values (circles) and trend line are showed. BDNF: brain-derived neurotrophic factor.

BDNF levels were significantly lower in patients (median, 1141 pg/ml; 25^th^ and 75^th^ percentiles, 1016 and 1704 pg/ml) than in healthy subjects (median, 2450 pg/ml; 25^th^ and 75^th^ percentiles, 2100 and 2875 pg/ml; *U* = 17, *p* < 0.001; Figure 1). They did not differ between patients with UWS (*n* = 10; median, 1480 pg/ml; 25^th^ and 75^th^ percentiles, 120 and 1972 pg/ml) and those in an MCS (*n* = 8; median, 1179 pg/ml; 25^th^ and 75^th^ percentiles, 833 and 1535 pg/ml; *U* = 26, *p* = 0.2) or between patients with TBIs (*n* = 8; median, 1314 pg/ml; 25^th^ and 75^th^ percentiles, 964 and 2029 pg/ml) and those with non-TBIs (*n* = 10; median, 1438 pg/ml; 25^th^ and 75^th^ percentiles 1064 and 1598 pg/ml; *U* = 33, *p* = 0.6; Figure 2). In the 12 patients who underwent ErigoPro treatment, BDNF levels measured before (median, 1438 pg/ml; 25^th^ and 75^th^ percentiles, 1068 and 1702 pg/ml) and after (median, 1141 pg/ml; 25^th^ and 75^th^ percentiles, 435 and 1680 pg/ml) treatment did not differ (*U* = 61, *p* = 0.5; Figure 3). BDNF levels correlated weakly with the time since brain injury (*r* = 0.53, *p* = 0.025; Figure 4), as they increased over time.

## 4. Discussion

In this study, serum BDNF levels were markedly reduced in patients with severe DOCs, to about half the levels found in healthy controls. Moreover, BDNF levels correlated positively with the time since brain injury. Finally, we found that the BDNF level was not modified by verticalization with robot-assisted stepping.

Neurotrophins such as BDNF may promote both neuronal survival and death, depending on different physiological and pathological conditions. Although mature BDNF promotes neurogenesis and activates prosurvival signaling through tropomyosin receptor kinases (Trks) [23–25], its precursor proBDNF binds to the p75 neurotrophin receptor (p75NTR), a tumor necrosis factor receptor-like molecule. Activation of p75NTR by proBDNF can induce neuronal apoptosis in different conditions [26–28]. p75NTR is expressed widely during synaptogenesis and subsequently downregulated in the adult brain. However, it is upregulated after brain injuries, including TBIs and cerebral hypoxia, and the resulting overexpression may cause neuronal death via different intracellular pathways in several conditions [29–32]. The reduction of serum BDNF levels in patients with UWS and those in an MCS may reflect a persistent impaired balance of interactions between BDNF and Trk and between proBDNF and p75NTR, as a consequence of brain injury. After severe brain injury, whether BDNF downregulation reduces neuronal death and apoptosis via less proBDNF–p75NTR binding or mainly impairs neurogenesis and synaptic plasticity via less mature BDNF–Trk binding remains unclear.

Other explanations can be offered for the reduction of serum BDNF levels in patients with DOCs following severe brain injury. In animal models of brain lesions, BDNF levels are increased in the cerebral tissue, in the hippocampus, and in the cerebral cortex [33, 34]. As compelling evidence suggests that BDNF crosses the blood–brain barrier bidirectionally [35–37], the reduction of serum BDNF levels in patients with DOCs may reflect increased use of circulating BDNF by the brain.

Another feasible explanation is that the reduction of the serum BDNF level is caused by the loss of BDNF-secreting neurons as a consequence of acute brain injury and subsequent brain atrophy. Neuroimaging studies have shown that patients with DOCs develop widespread cortical and subcortical atrophy [38–40]. Moreover, the degree to which markers of neuronal injury, such as neuron-specific enolase, are released is below normal in the chronic phases of DOCs [41], suggesting the progressive loss of neurons with brain atrophy [42]. Our results are in line with those of a previous study that documented reduced serum BDNF levels immediately postinjury until 12 months after TBI [43]. However, we also found that the reduction of serum BDNF does not remain constant over time; the BDNF level progressively tends to normalize, not supporting the argument that its reduction is due mainly to the loss of neurons with progressive brain atrophy. The physiological mechanisms that regulate BDNF levels over time in patients with DOCs remain still unknown, as do the clinical implications of changes in these levels.

Serum BDNF levels did not differ in patients with postacute DOCs of different etiologies in this study, suggesting that the reduction of the BDNF level in the postacute phase is not affected significantly by the mechanism of brain injury. Although few studies have evaluated BDNF levels in patients with nontraumatic brain lesions, the lack of difference according to the etiology of brain injury in this study is in agreement with the observation of reduced serum BDNF levels in the acute phase of stroke [44, 45]. In addition, no difference in the serum BDNF level between patients with UWS and those in an MCS was found. Both conditions are severe DOCs, and their clinical differentiation is often very difficult, leading to a high rate of misdiagnosis [46]. In this context, the difference in the BDNF level between patients with UWS and those in an MCS may be subtle, and the sample size in this study may have been inadequate to detect it.

This study failed to find changes in the serum BDNF level after verticalization associated with robot-assisted lower-limb training in patients with DOCs. To induce significant changes in the expression of BDNF, exercise must be sufficiently intense. Aerobic exercise has been associated clearly with increases in serum, plasma, and platelet BDNF levels immediately after exercise in healthy subjects [47, 48]. Under this condition, exercise is associated with changes in brain structure and functions that are believed to be BDNF mediated, such as increased hippocampus size and improved memory [49]. The aerobic threshold likely cannot be reached without voluntary exercise, and our results demonstrate that verticalization with passive stepping is not sufficiently intense to modify BDNF expression.

This study has several limitations. First, we did not determine the distribution of the common BDNF Val66Met polymorphism, which affects neuronal activity-dependent BDNF secretion [50], among patients and controls. Although we showed in a previous study that this polymorphism does not affect the outcomes of patients with posttraumatic UWS [51], we cannot exclude the possibility that its high frequency in patients in this study lowered their serum BDNF levels. Second, although we found that the serum BDNF level increased over time, we did not assess levels in the same patients at different times since brain injury; a more accurate evaluation could be performed in a longitudinal study. Third, we did not correlate BDNF levels with neuroimaging data; it is likely that BDNF levels can be affected by acute and chronic structural changes of the brain, such as loss of tissue, atrophy, or hydrocephalus. Finally, the parameters of the verticalization with robot-assisted stepping (i.e., tilt angle and number of steps per minute) were chosen arbitrarily; thus, we cannot exclude the possibility that different parameters, such as a greater tilt angle and more steps per minute, would induce significant changes in the BDNF level after exercise.

## 5. Conclusions

Serum BDNF levels are reduced in patients with DOCs, which may reflect decreased BDNF expression, increased cerebral binding, or reduced secretion. This reduction is not affected by DOC etiology or severity, and its magnitude decreases progressively as time since brain injury increases. BDNF plays central roles in synaptic plasticity and neuronal development, and animal models suggest that exogenous BDNF has neuroprotective effects, improving neurological deficits in different brain injuries, including TBI, hypoxic-ischemic brain injury, and subarachnoid hemorrhage [52–54]. Efforts to enhance BDNF expression with rehabilitative strategies are still in the early stage, and this study showed that verticalization with robot-assisted lower-limb training is inadequate for the promotion of BDNF-mediated plastic changes in patients with DOCs. Additional studies are needed to better understand the mechanisms underlying BDNF reduction after severe brain injury, to aid identification of the best rehabilitative strategies to promote restorative plastic changes and recovery in patients with DOCs.

## Figures and Tables

**Table 1:** Participants' details.

Variables	Patients (*n* = 18)	Healthy subjects (*n* = 16)
Males	10 (55.6%)	9 (56.3%)
Females	8 (44.4%)	7 (43.7%)
Age (years)	38.9 ± 17	38.9 ± 12.6
Time between brain injury and blood sample collection (months)	3.2 ± 2.2 (range 1–7.3)	
Etiology		
TBI	8	
Cerebral hypoxia	6	
Subarachnoid hemorrhage	2	
Brainstem hemorrhage	1	
Bilateral hemispheric ischemia	1	
Disorder of consciousness		
UWS (CRS-R score)^a^	4.5 ± 1.3	
MCS (CRS-R score)^a^	13.8 ± 3.6	

^a^CRS-R scores on the day of the blood sample collection. CRS-R: Coma Recovery Scale-Revised; MCS: minimally conscious state; TBI: traumatic brain injury; UWS: unresponsive wakefulness syndrome.

## Data Availability

Not available.
